# Similarities and differences between China and Sweden regarding the core features of palliative care for people aged 60 or older: a systematic scoping review

**DOI:** 10.1186/s12904-022-00906-7

**Published:** 2022-03-14

**Authors:** Gerd Ahlström, Hongli Huang, Yu Luo, Christina Bökberg, Birgit H. Rasmussen, Eva I. Persson, Lian Xue, Le Cai, Pingfen Tang, Magnus Persson, Jingjing Huang

**Affiliations:** 1 Department of Health Sciences, Faculty of Medicine, Lund University, P.O. Box 157, SE-221 00 Lund, Sweden; 2 Hospital Management, the Third People’s Hospital of Kunming, Kunming, Yunnan Province China; 3 Hospice Care Department, the Third People’s Hospital of Kunming, Kunming, Yunnan Province China; 4 The Institute for Palliative Care, Region Skane and Lund University, Lund, Sweden; 5 School of Public Health, Kunming Medical University, Kunming, Yunnan Province China; 6 The Medical Record Statistics Department, the Third People’s Hospital of Kunming, Kunming, Yunnan Province China

**Keywords:** Palliative care, Scoping review, Elderly, Older people, China, Sweden

## Abstract

**Background:**

Despite the increasing longevity of the world’s population, with an unprecedented rise in the number of people who need palliative care (PC), there has been sparse research regarding palliative care for older people, especially when it comes to comparison of PC between healthcare systems and cultures. The aim of this systematic scoping review was to identify the characteristics of the body of literature and to examine the knowledge gaps concerning PC research for older people (> 60 years) in two healthcare systems and cultures, mainland China and Sweden.

**Methods:**

The guidelines PRISMA (Preferred Reporting Items for Systematic Reviews), and PICOS (Patient/population, Intervention, Comparison/control, and Outcome) were used. Empirical studies on patients 60 years or older, next of kin or staff participating in a palliative care intervention or setting were included. They were conducted in mainland China or in Sweden during 2007–2019, were published in English and were extracted from seven databases: Embase, PubMed, Scopus, Cinahl, PsycInfo, Academic Search Complete and Cochrane Library. Two independent researchers conducted the selection of studies, data extraction and methodological evaluation. Any disagreements were resolved in consultation with a third researcher. The analysis was manifest directed content analysis based on PICOS domains.

**Results:**

Of the 15 studies, four were from mainland China and 11 from Sweden. Both countries included older patients with cancer but also other end-stage diseases such as heart failure and dementia. The studies differed in design, method and the content of the interventions. The study in China based on traditional Chinese medicine concerns traditional Chinese folk music. The six qualitative studies from Sweden were evaluations of five interventions.

**Conclusions:**

Despite the high age of the participating patients, there was no focus on an ageing perspective concerning palliative care. To adapt to the changes taking place in most societies, future research should have increased focus on older persons’ need for palliative care and should take account of issues concerning research ethics, ethnicity and culture.

**Registered in Prospero:**

CRD42020078685, available from.

**Supplementary Information:**

The online version contains supplementary material available at 10.1186/s12904-022-00906-7.

## Background

There is a speedily increasing need for palliative care (PC) in China and Sweden as a consequence of the fast-growing number of elderly in the population. This growth is a worldwide phenomenon, the estimate being that the global number of people aged 65 or more will have doubled to 1 in 6 by 2050, from 1 in 11 in 2019 [1]. The increased longevity means an increased need of PC since older people’s last years are commonly associated with such conditions as cardiovascular disease, chronic obstructive pulmonary disease, diabetes, cancer and dementia [2]. PC should be knowledge-based in order to meet the complex needs of older people, which is to say that it should be built on scientific research evidence, best clinical practice and the preferences of the patient, next of kin and healthcare professionals [2, 3].

At the same time the importance of local context and culture has increasingly been emphasised in healthcare, and this is highly relevant to end-of-life decision-making [3–5]. Chinese culture has developed from Taoism, Confucianism and Buddhism, which has influenced traditional Chinese medicine (TCM) [6]. However, Western medicine has had an increased influence on healthcare in China since the early twentieth century, although TCM still plays an important role and is planned to increase in the future [7, 8]. Swedish culture is based mainly on Christian traditions, even though Sweden today is relatively secular [9]. In a Western culture such as that of Sweden, embracing the TCM perspective with integration between mind and body is of increased interest in healthcare but involves cultural, legal and institutional challenges [10, 11]. To the best of our knowledge there has not been any research study that compares PC in the two healthcare systems and cultures, those of mainland China and Sweden.

Our research team has a long record of research collaboration between the two countries [12–15], and during this collaboration we have found that there is a lack of research comparing the different healthcare systems regarding PC. Such comparison, though, is of importance for staff competence and the provision of evidence-based care in these multicultural countries. To acquire a deeper understanding of the existing knowledge-gap we first consider systematic studies of PC in general (without focusing on ageing) published during 5 years and then, against this background, review empirical studies of PC interventions for older people, which is the focus of this research.

A summary of the general systematic review studies from China follows. One recently published review identified seven studies which together covered the following broad range of PC: home-based hospice (*n* = 1), inpatient hospice (n = 1), PC and nutrition support (n = 1), Advanced Care Plan (n = 1), family conferences in inpatient context (n = 1), music therapy (n = 1) and three-week review intervention (n = 1) [16]. This intervention for patients with terminal illness consisted of reviewing the person’s life and composing a life-review booklet. It was also identified in two systematic reviews of the effects of spiritual care on quality of life (QoL) and spiritual well-being. The main conclusion was that more rigorous designs should be used in future studies [17]. A meta-analysis of six randomised controlled trials (RCTs) from China revealed that music therapy improved the QoL of terminally ill patients, alleviating pain and psychological symptoms like anxiety and depression [18]. A review of 14 studies concerning Chinese herbal medicine (CHM) as a means of symptom management for people with cancer showed significantly reduced pain when conventional treatment was complemented by CHM [19]. A Cochrane review was based on 15 studies from China of people with severe or end-stage chronic kidney disease being provided with either haemodialysis or peritoneal dialysis. The effect of various types of acupuncture and related interventions was low and there was a high or unclear risk of bias in all these studies [20]. However, another review indicated that when conventional cancer care was complemented by acupuncture and related therapies there was an improvement in QoL, with reduced pain and fatigue [21]. In another review [22] a preliminary conclusion was that PC effectively relieves pain in patients with cancer. However, this review covered only a small number of studies (*n* = 18) with several methodological limitations and exhibited a high heterogeneity of both pharmacological and non-pharmacological treatments. In a review of six studies involving Qigong exercises for symptom management among cancer patients no form of Qigong was suggested as being superior to any another and the effectiveness was uncertain due to the limited number of Qigong trials, methodological problems and high risk of bias [23]. A systematic review concerning supervised walking 5 days a week during 3 weeks alleviated fatigue for patients with advanced-stage haematologic cancer [24]. To summarize, the identified studies were disparate in content and focus, which may illustrate the extremely limited access to palliative care for people living in China described in the literature [25, 26].

In Sweden, a broadly based literature review of PC research [27] found a large increase in the number of studies for the period 2007–2012 as compared with the period 1970–2006 (mean 44 per year for the six-year period as compared with four per year for the longer period). This review showed that still predominant were cross-sectional studies, qualitative and mono-disciplinary studies, with exclusion of ethnic minorities, nonverbally communicable people and the oldest people. Of the 263 studies for the period 2007–2012, only 4 % of the studies involved clinical interventions and only 1 % were implementation studies. This can be assessed as inadequacy when it comes to the development of knowledge-based PC. The interventions included palliative home care team, symptom management with pain treatment, soft tissue massage and non-pharmacological caregiving activities, artificial nutrition and hydration, creative activity, complementary and alternative medicine (CAM), [27]. To summarize, the number of intervention and implementation studies was unexpectedly small considering that PC was established in Sweden some 40 years ago and that the research has expanded steadily in volume [27].

The similarities found in the reviews from the two countries, not selected by age, were that PC studies mostly concern cancer, as the proportion of the non-cancer specific population was 8% in China [16] and 13% in Sweden [27]. However, studies from mainland China were somewhat more often included in systematic reviews of PC than those from Sweden. This is probably related to the fact that there were few intervention studies from Sweden [27].

There is an urgent need to acquire a more profound knowledge of PC for older people. The PC needs are increasing in the ageing populations worldwide, which means that professionals and researchers have to develop evidence-based PC. International collaboration may seem to be essential to enable healthcare professionals to better respond to cultural diversity in evidence-based practice [28]. The previous sparse research and the research group’s longstanding collaboration in practice and research [12–15] is the background to mapping the knowledge of PC for older people in China and Sweden. Therefore, the aim of this systematic scoping review was to identify the characteristics of the body of literature and to examine the knowledge gaps concerning PC research for older people (> 60 years) in two healthcare systems and cultures, mainland China and Sweden. With this in mind, the following three research questions were in focus; (1) What are the similarities and differences between the two countries regarding PC interventions for older people who are at the end of life?, (2) What does PC mean for older people from the perspective of different ethnic groups in each country?, and (3) What ethical questions were discussed in connection with palliative care for older people with incurable disease in each country?

## Methods

This study was a systematic scoping review based on the guidelines provided by PRISMA (Preferred Reporting Items for Systematic Reviews) [29]. A scoping review is a particularly appropriate method for identifying the body of evidence and knowledge gap when the literature is complex and heterogeneous [30, 31].

The study is registered in Prospero**,** CRD42020078685. Available from: https://www.crd.york.ac.uk/prospero/display_record.php?ID=CRD42020078685

### Eligibility criteria

A protocol including Patient/population, Intervention, Comparison/control, and Outcome **(**PICOS) was constructed (Table 1) in order to perform a systematic screening procedure in accordance with the aim of the study.

**Table 1:** Inclusion and exclusion criteria according to PICO used for studies about palliative care conducted in mainland China or Sweden

**Patient/Population**	
***Included if the article was about any of these***	
1. The older participants need to have an average age of 60 years or more and with expected limited time to live. The patients have needs of formal palliative care or informal care (efforts by next of kin).	
2. Next of kin of the older persons according to point 1.	
3. Staff can be included if it concerns interventions in palliative care including older persons according to point 1.	
No limitations on diagnosis, disease, comorbidity or sex.	
**Intervention/programmes**	
***Included any of the following studies***	
1. Interventions/activities for the older people in palliative care	
2. Actions/activities for next of kin in palliative care/family support	
3. Assessment methods used in palliative care	
4. Evaluation of needs, measures/efforts or activities in respect of the older people or their next of kin	
5. Organization of palliative care	
6. Ethnic groups/minorities	
7. Complementary methods or Traditional Chinese Medicine (TCM)	
8. Ethical consideration, dilemmas in palliative care	
9. Special forms of palliative care	
10. Collaboration and information transfer (between healthcare, municipality and/or authorities)	
**Comparison/control**	
Is there a comparison group when it is an RCT or an intervention?	
**Outcome measures**	
***Included if any outcome measure was studied, for example one of the following:***	
1. The quality of life of the older person or next of kin	
2. The participation in palliative care of the older person or next of kin	
3. Person-centred care	
4. Quality of care/Patient Satisfaction	
5. Ethical questions/dilemmas	
6. Side-effects (i.e. unwanted events, problems/difficulties/events associated with the intervention for the participants)	
7. Experiences	
8. Costs	
9. The above-mentioned outcomes that are distinguished by one or more of these aspects: gender, gender equality, ethnicity/culture or sexual orientation.	
**Exclusion criteria**	
1. Younger average age than 60 years.	
2. Studies not conducted in mainland China or Sweden.	
3. Languages other than English.	
4. Literature reviews, scoping and systematic reviews.	
5. Only theoretical studies.	
6. Not older patients’ or next of kin’s experiences of living with or staff’s experiences of working for a person who has a fatal or severe disease (when only focus on experiences, not the intervention in palliative care).	
7. Epidemiological studies on the prevalence of fatal diseases, mortality etc.	
8. Descriptive medical studies on disease only (eg stages of cancer). Also tests/testing of cancer drugs, chemotherapy and radiotherapy and other medical/surgical treatment methods.	
9. Psychometric studies alone.	
10. Study protocol	

To decide the period for the literature search a pilot test according to the inclusion and exclusion criteria (Table 1) was carried out in PubMed, which was the database expected to provide the greatest number of hits (see Additional file 1). There were 22 hits from China and 88 from Sweden. None of the 22 from China met the inclusion criteria: 15 were from Hong Kong or Taiwan, or included Chinese people living in other countries, six did not concern palliative care and one was not an intervention study (Additional file 1). Therefore the period chosen for inclusion was 1 January 2007 - 31 May 2019, this in order to allow comparisons between the two countries and also acquire deeper knowledge of palliative care interventions.

Regarding the 88 articles/hits found in the pilot test from Sweden, these showed the same pattern as reported in the previous review by the National Board of Health and Welfare, which included 133 studies for the same period up to and including 2006 [27]. Articles during that period consisted of predominantly qualitative design, small studies, cross-sectional studies and very few intervention studies.

Both quantitative and qualitative studies were included since there was no similar comparison study to be found in the Prospero register at the start of the study. Inclusion criterion were that the study was conducted in mainland China or in Sweden and have a published abstract. Furthermore, it has to be empirical studies involving interventions in palliative care or palliative settings of relevance for one of the included countries, and to have been published in a peer-review indexed journal. The patients were to be 60 years or older and with limited life-expectancy, and/or their next of kin or staff were included. The language was limited to English as this was the language shared by the involved researchers. More details of the inclusion and exclusion criteria are shown in Table 1.

### Databases and search strategy

Two information specialists and librarians from Lund University conducted a systematic search and screening procedure for identifying and excluding double hits (Additional file 2, Fig. 1). The following databases were examined: Embase, PubMed, Scopus, Cinahl, PsycInfo, Academic Search Complete and Cochrane Library. A first search was performed in September 2017 and a second in May 2019. The search terms were modified according to the specific vocabulary of the particular database. MeSH was used and the following terms (with “and/or”) were sought: Palliative care, palliative nursing, palliative therapy, Palliative medicine, Palliative treatment, Terminal care, Terminal illness, End-of life care, End-of life treatment, Hospice, Hospice care, Aged, Elderly, Over 60 years. After removal of duplicates the librarian delivered the references as an EndNote database (Clarivate Analytics, Philadelphia, PA, USA). Grey literature was not identified and included.

**Fig. 1:**
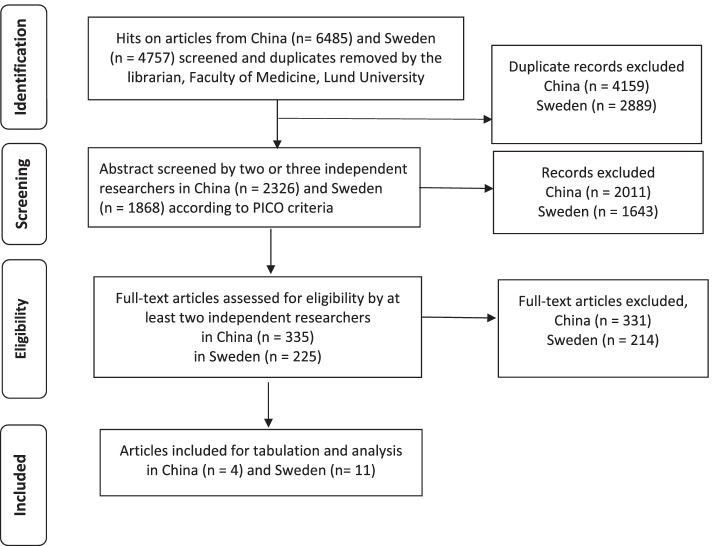
Flow chart of the identification and selection procedure of the articles according to Prisma guideline

### Study selection

All involved researchers had knowledge of the study aim and design before the start of the selection of studies, and the work was constantly discussed through Zoom online meetings (Zoom Video Communications, Inc.) or at face-to-face meetings chaired by the first author. The articles were stored and used in EndNote software. The steps for selection of the articles were based on PRISMA (Fig. 1) and PICOS. Two researchers independently reviewed the articles in each step. Any disagreements regarding study selection were resolved in consultation with a third or in some case fourth researchers. In the first step relevance was judged on the basis of abstract, thereafter on full text. The full text assessment was based on the selected EQUATOR guideline (Enhancing the QUAlity and Transparency Of health Research) [32] relevant to the design of the particular article. That means CONSORT for randomized trials, STROBE for observational studies, SRQR for qualitative research etc. The guidelines enabled the identification of the inclusion criteria in a similar manner independently of whether the researchers were from Sweden or China. The screening procedure resulted in 15 articles (Fig. 1).

### Data analysis

The analysis of quantitative and/or qualitative data in each article was in the form of directed content analysis [33] based on PICOS domains, and it was geared to similarities and differences between China and Sweden with regard to palliative care for older people. This manifest analysis was used to describe the existing body of literature, identifying study characteristics, the scope of what has been studied, context, the available findings and gaps that need to be filled [34]. The analysis of the text for each domain was done by two reviewers, one from each country, in an iterative model between the researchers (GA, JH), and discussions with the other co-authors were used to validate the finding. First, both researchers read the articles several times and did the tabulation (Tables 2 and 3). Second, a summary of each article was written based on PICOS domains. Third, the text was condensed from each article without interpretation. Fourth, the different condensed texts from all 15 articles were put together. Fifth, the essence of the whole was identified and presented in the results section concerning similarities and differences between China and Sweden with regard to palliative care for older people. The scales of the instruments were reviewed only in an overall perspective, not in focus for deeper analysis with regard to assessing the risk of bias in the included studies.

**Table 2:**
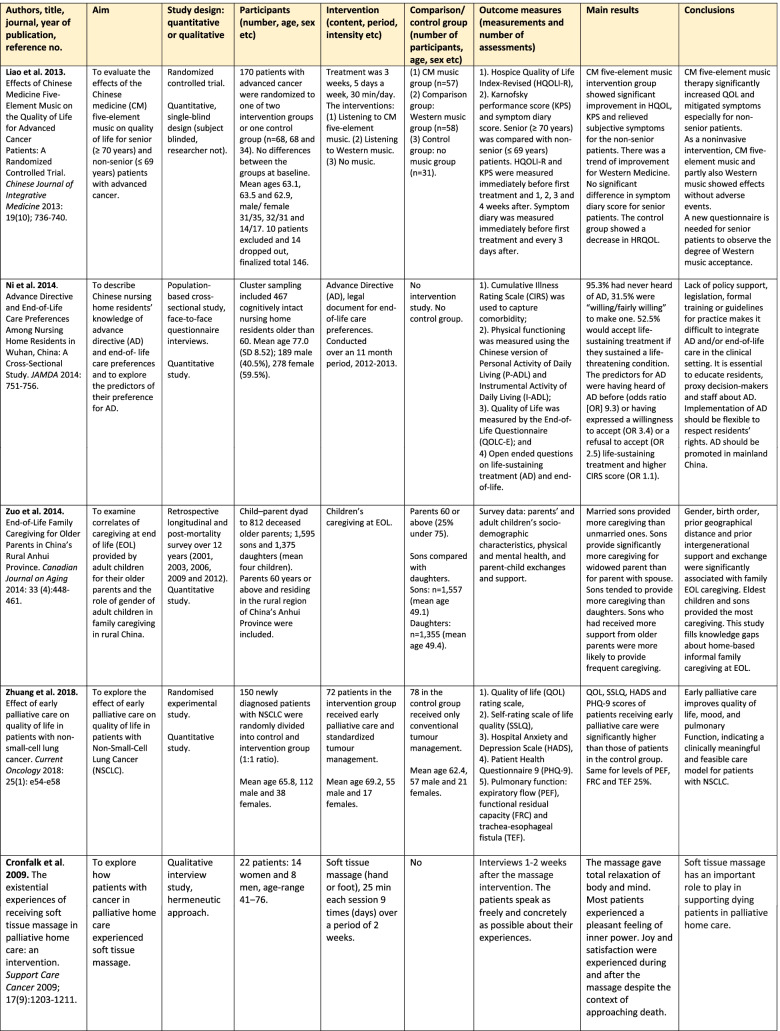

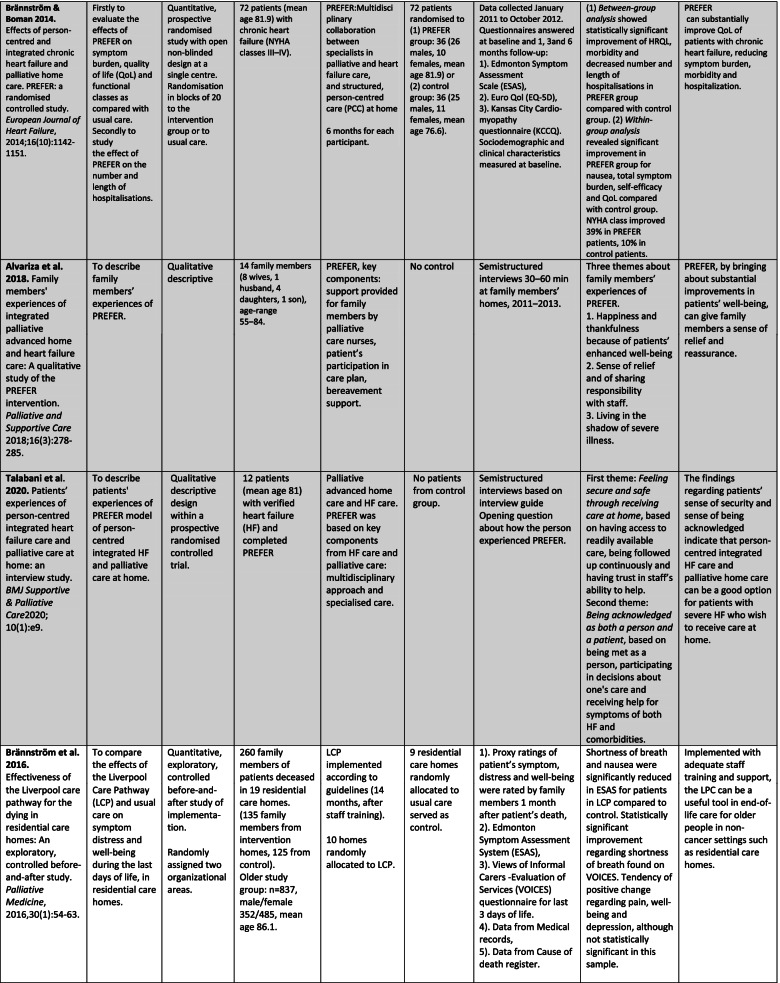

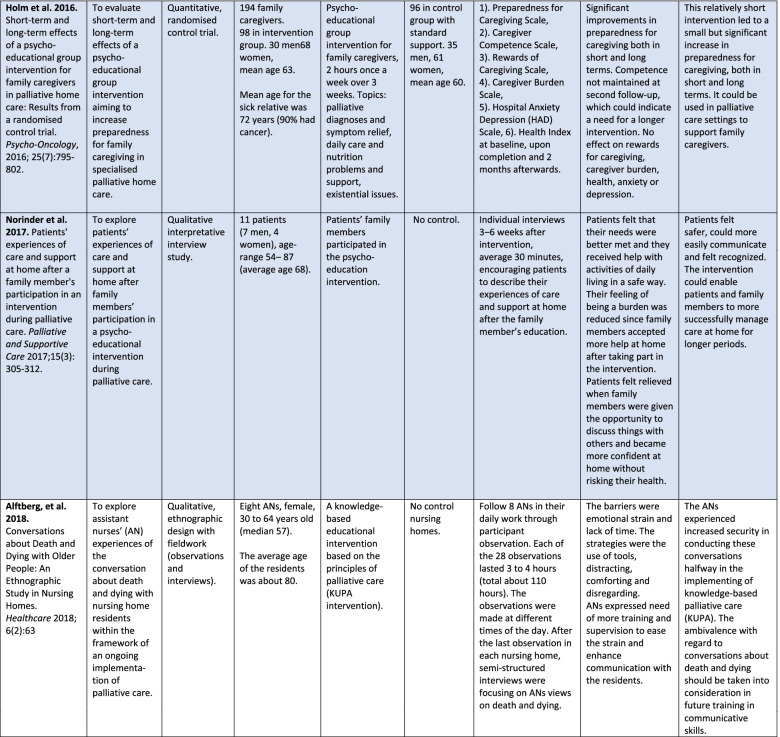

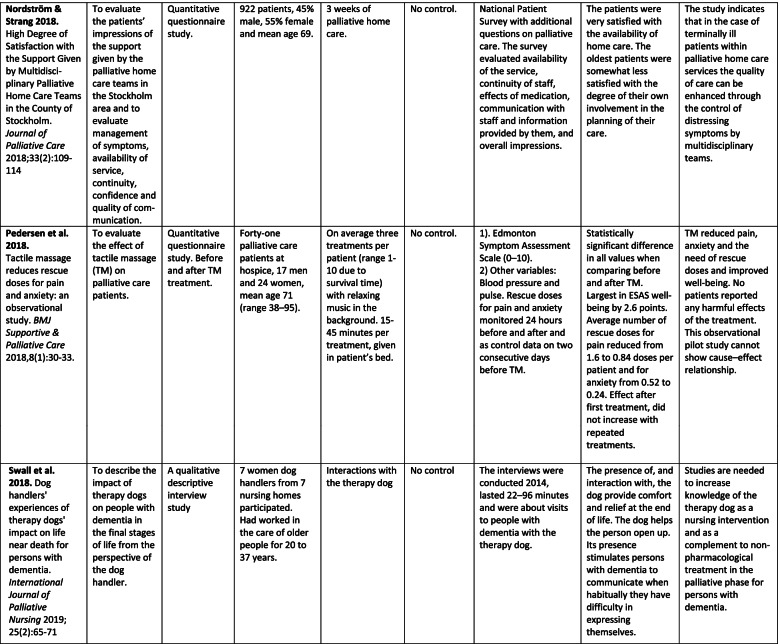
Overview of the studies (assessed as middle or high quality) about palliative care in China and Sweden. Studies highlighted in grey were carried out within the same project, those highlighted in blue within another

**Table 3:** The aim, study design and the findings of qualitative studies from Sweden (*n* = 6)

Aim of the study	Study design (participants and analysis)	Finding expressed by themes^a^ and categories/sub-themes
***Patients’ experiences***:		
To explore how patients with cancer in palliative home care experienced soft tissue massage (Cronfalk et al. [44]).	A qualitative interview study involving 22 patients with advanced cancer who received soft tissue massage. A hermeneutic approach was used in the analysis.	*Existential time of respite*. • An experience of thoughtful attention • A sensation of complete tranquility.
To describe patients’ experiences of PREFER model of person-centred integrated HF and palliative care at home (Talabani et al. [46]).	A qualitative descriptive interview study involving 12 patients with severe heart failure. Content analysis was used.	*Feeling secure and safe through receiving care at home.* • Having access to readily available care at home • Being followed up continuously and having trust in the team members’ ability to help. *Being acknowledged as both a person and a patient.* • Being met as a person, participating in decisions about one’s care • Receiving help for symptoms of both HF and comorbidities.
To explore patients’ experiences of care and support at home after family members’ participation in a psychoeducational intervention during palliative care (Norinder et al. [47]).	A qualitative interview study involving 11 patients with advanced incurable cancer. Interpretive descriptive analysis was used.	*Safe at home* *Facilitated and more honest communication* *Feeling like a unit of care.*
***Family’s experiences***:		
To describe family members’ experiences of the intervention, PREFER (Alvariza et al. [45]).	A qualitative descriptive design based on interviews with 14 members of the families of patients with chronic heart failure. Content analysis was used.	*Happiness and Thankfulness as a Result of Witnessing Patients’* *Enhanced Well-Being,* *Feelings of Relief and Shared Responsibility with Healthcare Professionals,* *Living in the Shadow of Severe Illness*.
***Staff’s experiences*:**		
To explore assistant nurses’ experiences of conversations about death and dying with nursing home residents within the framework of an ongoing implementation of palliative care (Alftberg et al. [49]).	An ethnographic study design was applied in seven nursing homes, where eight assistant nurses were interviewed and followed in their daily assignments through participant observations. An inductive thematic analysis was applied.	*Barriers to conversations about death and dying.* • Lacking time • Feeling emotional strain. *Managing conversations in practice.* • Having tools • Distracting • Comforting • Disregarding
To describe the impact of therapy dogs on people with dementia in the final stages of life from the perspective of the dog handler (Swall et al. [48]).	A qualitative interview study with 11 dog handlers for persons with dementia at seven municipal nursing homes. Qualitative content analysis was used.	*The presence of the dog and interaction with the dog provide comfort and relief at the end of life.* • The dog helps the person open up • The dog functions as a receiver and reliever • The dog is responsive and inspiring

^a^Themes are in italics

## Results

### Design of the studies included

Of the 15 articles included, four were from mainland China and 11 in Sweden (Table 2). Nine had a quantitative design, all four from China and five from Sweden. The remaining six were qualitative studies from Sweden.

Two of the Chinese studies were RCT studies [35, 36] and two were questionnaire studies [37, 38]. Two of the Swedish quantitative studies were RCT studies [39, 40], two were quasi-experimental studies, one with control group [41] and one without control group [42], and the fifth was a cross-sectional questionnaire study [43].

All of the six qualitative studies involved evaluations of RCTs or interventions [44–49]. Five of them were interview studies [44–48], the other one was an ethnographic study with participant observations, field notes and follow-up interviews [49]. In Sweden two large RCT projects were represented by three [39, 45, 46] and two articles respectively [40, 47].

The quantitative and qualitative results are presented integrated in the text and in Table 2. In addition, the results of the qualitative studies are presented separately in Table 3.

### Population

The number of participants varied greatly between the countries and study design (Table 2). Three of the four studies from China consisted of together 787 patients, whilst one included 2970 adult children of deceased older parents. Thus, the total number of participants from the Chinese studies were 3757 (range 170-2970). The 11 studies from Sweden involved 1811 participants (range 7-922): 1080 patients, 716 family members and 15 staff. Demographic data of the study groups such as mean age and sex were not comparable between the two countries.

Two of the studies from China focused on cancer [35, 36] while the other two did not focus on a specific disease. The respondents in the two non-cancer studies were the children of deceased older parents [38] and nursing home residents [37]. In Sweden only two of the 11 studies specifically involved patients with cancer [44, 47]. Three additional studies included patients in hospice care [42] and palliative home care [40, 43] without specific cancer focus even though that diagnosis is the most common in these types of care. Respondents in one of the studies concerning palliative home care were family members [40], while respondents in the other study were both patients and family members [43]. Three studies involved patients with chronic heart Failure (HF) [39, 45, 46]; in one of these studies the respondents were the family members [45]. Furthermore, one study used the handlers of therapy dogs as respondents and the patients were persons with dementia [48]. Finally, two studies concerned older patients without focus on specific diseases where the respondents were family members [39] and staff [49]. Both countries presented studies on older patients and cancer but Swedish studies have also included other end-stage diseases (HF and dementia), Table 2.

### Perspectives on palliative care

Some differences between the two countries concerning theoretical concepts of palliative care was found. The three earliest studies from China used the terms Chinese medicine [35] and end-of-life care [37, 38] but then in 2018 Zhuang and colleagues [36] gave a description of palliative care which is much in agreement with the Swedish perspective. Palliative care is seen as being based on interdisciplinary collaboration between staff and is designed to alleviate symptoms, promoting the patient’s and the family’s QoL and facilitating the staff’s communication with the family. All studies from Sweden [39–49] used palliative care as the main concept and the term end-of-life only as a description of the late life phase. Some studies from Sweden focused on person-centred care and integrated care as new concepts for describing palliative care [39, 45, 46], but these concepts were not mentioned in the studies from China.

Another difference found between the countries was that Chinese culture was reported as influencing the development of palliative care in China, whilst no influence of culture was reported in the case of Sweden. The barriers mentioned in the Chinese studies were that death is a very sensitive issue, a topic people avoided speaking about, and that filial piety implies children’s fundamental responsibility for providing care for an ageing parent nearing the end of life [37, 38].

### Interventions and exploration of palliative care

The RCT studies were not blinded for the participants or the health professionals. Two randomised intervention studies were from China, one evaluated the effect of the TCM five-element music on QoL for senior and non-senior patients with advanced cancer [35], the other the effect of early palliative care on QoL in patients with non-small-cell lung cancer (NSCLC) [36]. Of the two cross-sectional studies from China, one evaluated the family caregiving pattern at end-of-life [38], the other evaluated end-of-life care preferences in the form of the Advance Directive (AD) for nursing home residents [37].

One of the two randomised intervention studies from Sweden evaluated the intervention PREFER (Palliative advanced home caRE and heart failurE caRe). The target group were patients with HF and the intervention was based on key components in this cardiac care and specialised palliative care, person-centred, team-based, home-based and structured care [39]. This programme was evaluated by patients [39] and family members [45, 46]. The second randomised intervention study also concerned palliative home care but was a psycho-educational group intervention for family caregivers, evaluated on family caregivers and patients [40, 47].

The other interventions comprised massage [42, 44], the structured standard care plan known as the Liverpool Care Pathway (LCP) [41], palliative care education for the staff at nursing homes [49], multidisciplinary palliative home care teams [43], and use of therapy dog for patients with dementia [48]. These interventions were evaluated by patients [42–44], family members [41, 43], and staff [48, 49], Table 2.

### Control groups in the studies

The two RCT studies in China were designed with randomised control groups and three of the RCT studies in Sweden involved control groups, as shown in Table 2.

### Outcome measures

QoL and symptom assessment were the most common primary outcome measures when evaluating interventions. The interventions evaluated quantitatively (Table 2) use a number of different validated questionnaires (*n* = 16). Of these, the Hospital Anxiety and Depression Scale (HAD) was used in both countries [36, 40]. The Edmonton Symptom Assessment Scale (ESAS, which measures pain, tiredness, nausea, depression, anxiety, drowsiness, appetite, well-being, and shortness of breath) was used in three Swedish interventions [39, 41, 42], but none from China. Independently of which intervention programme was used, all evaluations showed enhanced QoL and decreased symptom burden in the intervention group post-intervention as compared with before and/or in comparison with a control group.

Table 3 shows the results from the six qualitative studies in terms of themes, sub-themes and categories. The qualitative studies are all from Sweden, none is from mainland China. The results provide greater understanding of each separate intervention in palliative care from the perspectives of patients (*n* = 3), family members (n = 1), assistant nurses and handlers of therapy dogs working in nursing homes (*n* = 2).

## Discussion

The results show that palliative care interventions concerning older people were scarce since only 15 of totally 4194 identified articles at the start were included in this scoping review (the inclusion rate in China was 0.17% and in Sweden 0.59%). However, several studies in both countries were excluded because of not fulfilling the inclusion criteria. The main reasons for exclusion in China were that the interventions were medical palliative treatments (not palliative care, were not conducted in mainland China (the sample were instead Chinese immigrants living and receiving palliative care in other countries such as the USA) or were not focused on older people. The main reasons in Sweden were no intervention, not concerning older people or deficient information on the average age of the study group. The only TCM study in China was about music, whilst in Sweden there were two studies on massage but these were not reported as being based on a TCM perspective. In both countries symptom and QoL assessments were applied in the evaluations. However, the only instrument used in both countries was the HAD-scale for anxiety and depression. The six qualitative articles included the perspectives of patients, family and staff. To summarize, the included studies from each country differ from each other in respect of number of studies, designs and methods, and in the content of the interventions.

It is well-known that old age is strongly associated with a high risk of frailty and multimorbidity (two or more chronic health conditions in an individual) [50–52]. According to the WHO report on palliative care for older people [2], the specific needs in the end-of-life phase have received too little focus despite the fact that death is most common among older people. Palliative care should be provided more widely and become integrated into all relevant health care, not only offered to people with cancer in hospice or specialist units [2]. Despite an average age of 60 years or older in the included studies, only a few mentioned briefly the impact of age on the design of the palliative care intervention [48, 49]. There is a significant need to develop palliative care to meet the complex needs of older people due to multimorbidity and the sharply increased number of older people in the world including China and Sweden. One way is to broaden the competence within the palliative care team with a geriatrician and a nurse with special training in geriatric care to complement the oncology competence area [2, 53].

Research about palliative care produces various ethical concerns and methodological complexities since it involves vulnerable dying people. All studies in this review had been rigorously reviewed by the relevant ethical authority for each country. One common threat to the quality of the results in studies involving people with life-threatening conditions is high heterogeneity due to varied prognosis for different diseases, another is high attrition [2, 27, 54]. Development of new interventions or adaptation of established interventions for new groups such as older people presupposes evidence before implementation in daily palliative care. However, the need for new knowledge must be weighed against the risk of harm and the right not to be exposed to tiring research in a vulnerable situation without informed consent [54, 55]. A few studies from Sweden in this review reported ethical concerns; none from China did so. Cronfalk and colleagues [44] consider that the intervention with massage in the patient’s home involves a risk of intrusion upon integrity, whilst the human interaction in connection with massage can threaten the effect of this intervention. The decision to rely on proxy raters in research such as family members [41] and dog-handlers instead of the patients in the last phase of palliative care was taken in order to ensure nonmaleficence [48]. In contrast, Cronfalk and colleagues (2009) found that patients wanted to participate in the late phase of palliative care in order to contribute to the development of knowledge. Altruism was the main finding in a review of ten empirical studies concerned with dying persons’ attitudes to participating in research. It was meaningful for them to help others in a similar situation, to help society and to contribute to the progress of knowledge. However, the willingness to participate differed between designs and of study and in accordance with the degree of effort required of the patient [56]. Research ensures that palliative care provided for dying persons continues to become developed and evidence-based [2, 56]. Still, such research needs to be conducted with sensitivity, respecting the person’s autonomy and dignity [54, 55].

A systematic review of 50 articles demonstrates that older people had varied views concerning life and death, commonly involving a pragmatic acceptance of death’s inevitability [57]. Cultural beliefs and values are critical for people’s preferences and how they manage end-of-life. Culture can be described as a complex, multifaceted phenomenon shaped by interactions between socio-demographic factors and continuous processes of redefinition deriving from historical experiences and social realities [5]. In almost all developed and some developing countries, palliative care (like other forms of healthcare) has increased in complexity at the same time as the work of healthcare professionals is expected to be evidence-based. For evidence to be successfully implemented into practice, it is essential that the issues of context and cultural be considered. By critically reflecting on taken-for granted assumptions a greater understanding of both practice and the evidence available for use in practice can be acquired [58]. Knowledge and awareness of culture are therefore a necessity for professionals in palliative care who are to meet the needs of dying patients and their families of different cultural descent [5, 6]. In this review, three of the four Chinese studies described Chinese culture as a background to the study [35, 37, 38], but none of the Swedish studies takes account of cultural aspects.

Zuo and colleagues [38] investigated the children’s provision of the parents’ end-of-life care in rural areas. In the rural part of China end-of-life care is primarily provided by the family since there is little access to community-based palliative care. The results showed that the traditional shouldering of responsibility by the eldest sons and their spouses, based on the principles of Confucianism, was being transferred to eldest daughters, who increasingly provided the care of their parents. This fact was to be explained by the changing economy and culture in the context of the mass migration of young adults from rural areas [38]. In contrast to Swedish culture, the relatives’ power is more important than patient autonomy, which means that older Chinese people often prefer that their family make all care or treatment decisions [37].

Another cultural difference between the two countries concerns the patterns of communication with regard to dying and death [5]. The interventions from Sweden in respect of person-centred and integrated care [39, 45, 46] and in respect of education [40, 47, 49] indicate the vital role communication has in palliative care. The goal of this communication is to create sensitive, open and safe palliative care [43]. Studies from China show that death is a taboo subject that generates fear of bad luck [6, 25, 37]. The consequence is avoidance of communication about death by staff and family members [37], which may contribute to the concentration on striving to prolong the patient’s life as long as possible [6]. The problem is that patients may misunderstand their illness and prognosis and have no time to say goodbye, which is negative both for themselves and for their families [59]. There is need for further investigation of how cultural aspects shape meaning with regard to life and death and influence decision-making at the end of life.

This scoping review provides a basis for future research projects that can bridge the existing knowledge gap concerning PC, which is of considerable importance when it comes to meeting the needs of patients representing European and Chinese cultures. The identified areas could appropriately be the focus of future systematic reviews or other types of evidence synthesis.

### Methodological considerations

This review has three limitations. Firstly, the studies had to be published in the English language. Even though palliative care has recently become an increasing area of healthcare in China [25, 26, 59], many studies on TCM are published in Chinese and therefore not included in this study. However, the literature reviews of palliative care interventions for patients of adult age (without focus on older people, summarized in the Introduction), indicate that these studies were small and without a control group and therefore did not fit the quality criteria for a systematic review. Scoping reviews are particularly helpful when the literature is complex and heterogeneous. It is also appropriate to assess and understand the extent of the knowledge in an emerging field or to identify, map, report or discuss the characteristics or concepts in that field [30, 31]. A recent scoping review of cohort studies in Chinese and English shows that TCM interventions have increased rapidly but the studies were of poor quality [60]. This limitation needs to be in mind when interpreting the results. A systematic review is to be recommended in the future to evaluate the evidence emerging from the steadily increasing number of intervention studies and to address any uncertainty or variation in their designs [30, 31].

Secondly, the studies included in this review revealed a significant variation in number, designs and focus of research questions and methods, when comparison was made within each country but especially when it was made between the countries. Given this diversity, no meta-analysis or meta-synthesis was undertaken. Instead, we used the PICO domains as the structure for the analysis. The final textual analysis was performed by two authors, one from each country (GA and JJ), and validated by the others. The selection of the studies was based on independent reviews of articles made according to Prisma by at least two researchers [29]. When there were conflicting results, three or sometimes four researchers reviewed the study in question until a final decision was made. The quality assessment was based on EQUATOR guidelines [32], and the guideline used was adapted to the particular design of the study. Furthermore this guideline is comprehensive and the disagreements were often caused by the reviewers’ varied focus in respect of quality criteria. From such disagreements there emerged the best possible judgement with an additional reviewer.

Thirdly, the intervention studies were so few in number that we had to broaden the design to include at least a few studies that contribute to knowledge about palliative care in the particular country. This weakness needs to be kept in mind when interpreting the results of this study. In the light of this we see the value of doing a follow-up study over the next decade to acquire more evidence-based knowledge and learn more from each other’s countries about palliative care.

A strength in this study was the pilot test of the literature search which resulted in a time limitation starting from 2007. This was a way of achieving reliability and efficiency and avoiding publication bias in the literature search by the information specialists/librarians [61].

## Conclusions

This scoping review revealed very few palliative care interventions for people 60 years and above in both mainland China and Sweden. The differences were in the number of studies, in their perspective, design and method, and in the content of the interventions. It is therefore unclear whether the interventions are in line with the specific needs of older patients and their family members. Learning from each country regarding evidence-based interventions is limited in this step due to the small number of interventions, considerable variation and unsatisfactory scientific quality. This review represents an initial step in research about PC interventions in these two different healthcare systems and cultures. Palliative care is in an expansive phase, especially in China, making it of interest to carry out a systematic review in a follow-up study in the coming decade to evaluate whether the intervention research and practice is based on the established quality of evidence. Future research should have increased focus on older persons’ need for palliative care and should take account of issues concerning research ethics, ethnicity and culture.

## Supplementary Information

**Additional file 1.** Identified articles from mainland China and reason of exclusion in the pilot test for all years up to 2007 before the main systematic literature.

**Additional file 2.** Literature search for China and Sweden.

## Data Availability

The strategies of the literature searches are presented in the Additional files 1 and 2. The results are based on articles that can be found in, and downloaded from, the PubMed database or any of the others used: Embase, Scopus, Cinahl, PsycInfo, Academic Search Complete and Cochrane Library.

## Abbreviations

CAM: Complementary and alternative medicine
CHM: Chinese herbal medicine
EQUATOR guidelines: Enhancing the QUAlity and Transparency Of health Research
HAD: The Hospital Anxiety and Depression Scale
PC: Palliative care
PICOS: Patient/population, intervention, comparison/control, and outcome
PRISMA: Preferred reporting items for systematic reviews
QoL: Quality of life
RCT: Randomised controlled trials
TCM: Traditional Chinese medicine
