# Supplementary material for: Similarities and differences between China and Sweden regarding the core features of palliative care for people aged 60 or older: a systematic scoping review
Source: BMC Palliat Care. 2022 Mar 14;21:35. doi: 10.1186/s12904-022-00906-7 (PMC8922883; doi:10.1186/s12904-022-00906-7)
Supplement: Supplementary file 1 — Additional file 1. Identified articles from mainland China and reason of exclusion in the pilot test for all years up to 2007 before the main systematic literature. [file 12904_2022_906_MOESM1_ESM.docx]

**Additional file 1.** Identified articles from China and reasons for exclusion of the articles in the pilot test for all years up to 2007.

**Search terms for China (N=22):** (((((("palliative care"[MeSH Terms]) OR (terminal care[MeSH Terms]))) OR (hospice and palliative care nursing[MeSH Terms])) OR (hospice care[MeSH Terms])) AND ((aged[MeSH Terms]) OR (frail elderly[MeSH Terms]) OR "over 60")) AND (china[MeSH Terms] OR China[Title]) AND (1982:2006[pdat]) NOT hong kong[Title]. Filters: Abstract, English.

**Search terms for Sweden (N=88):** (((((("palliative care"[MeSH Terms]) OR (terminal care[MeSH Terms]))) OR (hospice and palliative care nursing[MeSH Terms])) OR (hospice care[MeSH Terms])) AND ((aged[MeSH Terms]) OR (frail elderly[MeSH Terms]) OR "over 60")) AND (sweden[MeSH Terms] OR Sweden[Title]) Filters: Abstract, English, from 1980 – 2006. The results from Sweden are mentioned only on page 7 of the text in the manuscript.

| **Identified articles from China (n=22)** | **Reasons for exclusion of the articles in the pilot test (n=22)** | | |
| --- | --- | --- | --- |
| **Authors, title, journal, year** | **Not palliative care** (n=6) | **Palliative Care but no intervention** (n=1) | **Not mainland China**  (n=15) |
| Lewis M. [Aging in the People's Republic of China.](https://pubmed.ncbi.nlm.nih.gov/7183562/) *Int J Aging Hum* *Dev* 1982. |  |  | Insights gained regarding aging in China through discussion with a number of Chinese living in the United States and Hong Kong, experts and a review of literature. |
| Hu P. [The acceptability of active euthanasia in China.](https://pubmed.ncbi.nlm.nih.gov/8377620/) *Med Law* 1993. | Theoretical discussion of the concept of euthanasia with reference to two cases. |  |  |
| Gertsch P, Yip SK, Chow LW, Lauder IJ. [Free perforation of gastric carcinoma. Results of surgical treatment.](https://pubmed.ncbi.nlm.nih.gov/7531430/)  *Arch Surg* 1995. |  |  | Results of treatment of free perforation of gastric carcinoma in a consecutive number of patients treated at institution in Hong Kong. |
| Yuen AP, Wei WI, Hui Y, Ho WK. [Comprehensive analysis of pharyngeal recurrence of laryngeal carcinoma after total laryngectomy.](https://pubmed.ncbi.nlm.nih.gov/8944296/)  *Am J Otolaryngol* 1996. | Review of the records of patients who had undergone total laryngectomy for the treatment of squamous cell carcinoma of the larynx. |  | (Hong Kong, additional reason for exclusion). |
| Wang XS, Mendoza TR, Gao SZ, Cleeland CS.  [The Chinese version of the Brief Pain Inventory (BPI-C): its development and use in a study of cancer pain.](https://pubmed.ncbi.nlm.nih.gov/8951936/) *Pain* 1996. |  | Psychometric study of a Chinese version of the Brief Pain Inventory (BPI-C) and its reliability and validity. |  |
| Sze FK, Chung TK, Wong E, Lam KK, Lo R, Woo J.  [Pain in Chinese cancer patients under palliative care.](https://pubmed.ncbi.nlm.nih.gov/9743825/) *Palliat Med* 1998. |  |  | Assessment of the prevalence, nature and intensity of pain in Chinese patients with advanced cancer and their characteristics (Hong Kong). |
| Lo RS, Ding A, Chung TK, Woo J. [Prospective study of symptom control in 133 cases of palliative care inpatients in Shatin Hospital.](https://pubmed.ncbi.nlm.nih.gov/10659102/) *Palliat Med* 1999. |  |  | Study of the prevalence and severity of physical and nonphysical symptoms, and the benefits from treatment, in advanced cancer patients presenting to a palliative care unit (Hong Kong). |
| Yeung EW, French P, Leung AO. [The impact of hospice inpatient care on the quality of life of patients terminally ill with cancer.](https://pubmed.ncbi.nlm.nih.gov/10526428/) *Cancer Nurs* 1999 |  |  | Exploration of the needs and concerns of patients with terminal cancer in a hospice and evaluation of patients’ QoL (Hong Kong). |
| Liu JM, Lin WC, Chen YM, Wu HW, Yao NS, Chen LT, Whang-Peng J.  [The status of the do-not-resuscitate order in Chinese clinical trial patients in a cancer centre.](https://pubmed.ncbi.nlm.nih.gov/10461593/) *J Med Ethics* 1999. |  |  | Descriptive analysis of the pattern of end-of-life decision-making for terminal Chinese cancer patients (Taiwan). |
| Low JA, Ng WC, Yap KB, Chan KM. [End-of-life issues--preferences and choices of a group of elderly Chinese subjects attending a day care centre in Singapore.](https://pubmed.ncbi.nlm.nih.gov/10748965/) *Ann Acad Med Singap* 2000. |  |  | Descriptive analysis of the choices and preferences of a group of elderly Chinese subjects attending a day care centre in Singapore with regard to end-of-life issues. |
| Bowman KW, Singer PA.  [Chinese seniors' perspectives on end-of-life decisions.](https://pubmed.ncbi.nlm.nih.gov/11459396/) *Soc Sci Med* 2001. |  |  | Qualitative survey of attitudes towards end-of-life decision-making in the case of seniors at a Chinese community centre in Toronto, Canada. |
| Mak MH. [Awareness of dying: an experience of Chinese patients with terminal cancer.](https://pubmed.ncbi.nlm.nih.gov/12542081/) *Omega* 2001. |  |  | Inquiry directed towards gaining an understanding of what it means to die a "good death" from the perspective of Chinese patients (Hong Kong). |
| Law WL, Chan WF, Lee YM, Chu KW. [Non-curative surgery for colorectal cancer: critical appraisal of outcomes.](https://pubmed.ncbi.nlm.nih.gov/14618348/)  *Int J Colorectal Dis* 2004. | Evaluation of outcomes in patients undergoing non-curative surgery for colorectal cancer and identification of patients who would benefit from palliative surgery. |  |  |
| Mok E, Chiu PC.  [Nurse-patient relationships in palliative care.](https://pubmed.ncbi.nlm.nih.gov/15533085/)  *J Adv Nurs* 2004. |  |  | Exploration of the nurse–patient relationship in the context of palliative care (Hong Kong). |
| Wong FK, Liu CF, Szeto Y, Sham M, Chan T.  [Health problems encountered by dying patients receiving palliative home care until death.](https://pubmed.ncbi.nlm.nih.gov/15238813/)  *Cancer Nurs* 2004. |  |  | Prospective study of the health problems encountered by dying patients receiving home care, from referral to home care until death (Hong Kong). |
| Tse CY, Tao J. [Strategic ambiguities in the process of consent: role of the family in decisions to forgo life-sustaining treatment for incompetent elderly patients.](https://pubmed.ncbi.nlm.nih.gov/15371188/) *J Med Philos* 2004. |  |  | Evaluation of the Hong Kong approach to consent regarding the forgoing of life-sustaining treatment for incompetent elderly patients. |
| Woo PC, Teng JL, Leung KW, Lau SK, Wong MK, Yuen KY. [Bacteremia in a patient with colonic carcinoma caused by a novel Sedimentibacter species: Sedimentibacter hongkongensis sp. nov.](https://pubmed.ncbi.nlm.nih.gov/15474315/)  *Diagn Microbiol Infect Dis* 2004. | Review of systemic therapy with radionuclides used for patients with painful skeletal metastases, from the point of view of its efficacy, cost and level of toxicity. |  |  |
| Lam PT, Chan KS, Tse CY, Leung MW. Retrospective analysis of antibiotic use and survival in advanced cancer patients with infections, *J Pain Symptom Manage* 2005. | Review of the pattern of use of antibiotics and identification of factors that could affect outcomes after infection. |  |  |
| Chan J, Kayser-Jones J. [The experience of dying for Chinese nursing home residents: cultural considerations.](https://pubmed.ncbi.nlm.nih.gov/16130359/)  *J Gerontol Nurs* 2005. |  |  | Study concerning cultural diversity of the Chinese elderly population in the United States. |
| Chan CW, Richardson A, Richardson J. [A study to assess the existence of the symptom cluster of breathlessness, fatigue and anxiety in patients with advanced lung cancer.](https://pubmed.ncbi.nlm.nih.gov/16298550/)  *Eur J Oncol Nurs* 2005. | Small-scale study to assess the existence of symptom cluster involving breathlessness, fatigue and anxiety in patients with advanced lung cancer undergoing palliative radiation. |  | (Hong Kong, additional reason for exclusion). |
| Lee WT, Chan HF, Wong E. [Improvement of feeding independence in end-stage cancer patients under palliative care: a prospective, uncontrolled study.](https://pubmed.ncbi.nlm.nih.gov/16133073/) *Support Care Cancer* 2005. |  |  | Study of effectiveness of occupational therapy in promoting feeding independence in end-stage cancer patients in palliative care (Hong Kong). |
| Yan S, Kin-Fong C. [Quality of life of patients with terminal cancer receiving palliative home care.](https://pubmed.ncbi.nlm.nih.gov/17263052/) *J Palliat Care* 2006 |  |  | The levels of quality of life of patients with terminal cancer who received palliative care in home settings (Hong Kong). |
