# Supplementary material for: Similarities and differences between China and Sweden regarding the core features of palliative care for people aged 60 or older: a systematic scoping review
Source: BMC Palliat Care. 2022 Mar 14;21:35. doi: 10.1186/s12904-022-00906-7 (PMC8922883; doi:10.1186/s12904-022-00906-7)
Supplement: Supplementary file 2 — Additional file 2. Literature search for China and Sweden. [file 12904_2022_906_MOESM2_ESM.docx]

**Additional file 2.** Literature Search for China and Sweden

# Project KIT (Key Intelligent topic)/Client

| Reportdate | Projectname | CLIENT | Prepared by |
| --- | --- | --- | --- |
| 23 May 2019 | Updated systematic scoping review on palliative care in Sweden and China | Gerd Ahlström | MB&CT |
| 9 September 2017 | Systematic scoping review on palliative care in Sweden  and China | Gerd Ahlström | MB&CT |

# Project KIQ (Key Intelligent Questions) – Project Decriptions

The aim of this systematic scoping review is to identify, analyse and compare palliative care for older people in two healthcare systems, Sweden and China.

- What are the similarities and differences in the two countries regarding palliative care interventions for older people with known limited time to live or who are at the end of life?
- What means palliative care for older people from the perspective of different ethnic groups in each country?
- Which ethical questions are discussed in connection with palliative care for older people with incurable disease?

# Project Keywords

Palliative care, palliative nursing, palliative therapy

Palliative medicine, Palliative treatment

Terminal care, Terminal illness

End-of life care, End-of life treatment

Hospice, hospice care

Aged, elderly, over 60, over 80

# Project ISS (Information Service Sources)

PubMed, Embase, Scopus, PsycInfo, Cochrane Library, Academic Search Complete, Cinahl Complete

# Database Searches /search strings

**Update searches: 2017-2019**

|  | **Sweden** | **China** |
| --- | --- | --- |
| Embase | 382 | 725 |
| PubMed | 305 | 362 |
| Scopus | 129 | 260 |
| Cinahl | 67 | 60 |
| PsycInfo | 57 | 57 |
| Academic Search Complete | 197 | 272 |
| Cochrane Library | 13 | 24 |
| Total | 1150 | 1770 |
| After deduplication | 576 | 1146 |

**Original searches: 2007-2017**

|  | **Sweden** | **China** |
| --- | --- | --- |
| Embase | 1040 | 1440 |
| PubMed | 813 | 1233 |
| Scopus | 388 | 662 |
| Cinahl | 292 | 245 |
| PsycInfo | 436 | 351 |
| Academic Search Complete | 614 | 715 |
| Cochrane Library | 24 | 69 |
| Total | 3607 | 4715 |
| After deduplication and removal of irrelevant records | 1292 | 1180 |

**Updated Literature Searches – Sweden Palliative Care: Search strategies Sept 2017-2019**

**Embase Update SWEDEN, 20170905 - 20190523**

#1 'sweden'/exp OR sweden OR Swedish 702,053

#2 'palliative nursing'/exp OR 'palliative therapy'/exp OR 'terminal care'/exp OR 'hospice'/exp OR 'hospice care'/exp OR palliative OR 'terminal illness' OR 'palliative care' OR 'palliative treatment' OR 'palliative medicine' OR 'terminal care' OR 'end of life care' OR 'end of life treatment' OR hospice OR hospices OR 'palliative intervention' OR 'end of life care'/exp OR 'bereavement care' OR 'end of life' 230,532

#3 #2 OR 'terminally ill patient'/exp OR 'terminally ill' 233.040

#4 aged OR elderly OR older OR 'over 60' OR 'over 60' OR 'over 80' OR frail OR senior OR 'very old' OR 'old age' OR geriatric OR oldest OR elder OR 'end of life' OR 'late stage' OR 'palliative care' OR 'aged'/exp 4,760,689

#5 #1 AND #3 AND #4 2,609

#6 #5 [english]/lim AND [1-9-2017]/sd NOT [2-6-2019]/sd 551

#7 #6 AND ('article'/it OR 'article in press'/it OR 'review'/it) 382

**PubMed Update SWEDEN, 20170905-20190523**

#1 "Palliative Care"[Mesh] OR "Palliative Medicine"[Mesh] OR palliative OR "terminal illness" OR "Terminal Care"[Mesh] OR "Hospice Care"[Mesh] OR "Hospices"[Mesh] OR "Hospice and Palliative Care Nursing"[Mesh] OR "palliative care" OR "palliative treatment" OR "palliative medicine" OR "terminal care" OR "end of life care" OR “end of life treatment” OR hospice OR hospices OR "palliative intervention" OR “terminally ill”[Mesh] OR “terminally ill” OR “end of life” OR “bereavement care” 148,948

#2 ("Sweden"[Mesh] OR Sweden OR Swedish)) 383,566

#3 aged OR elderly OR older OR “over 60” OR “over 60” OR “over 80” OR frail OR senior OR “very old” OR “old age” OR geriatric OR oldest OR elder OR “end of life” OR “late stage” OR “palliative care” OR “aged”[Mesh] 5,405,717

#4 #1AND #2 AND #3 1,776

Filters: Publication date from 2017/09/01 to present; English 323
Filter: journal article, review 305

## **Scopus Update SWEDEN, 20170906 - 20190524** #1 TITLE-ABS-KEY ( sweden  OR  swedish )  204,182

#2 TITLE-ABS-KEY  (“palliative nursing” OR “palliative therapy” OR “terminal care” OR hospice OR “hospice care” OR palliative OR “terminal illness” OR “palliative care” OR “palliative treatment” OR “palliative medicine” OR “end of life care” OR “end of life treatment” OR hospices OR “palliative intervention” OR “bereavement care” OR “end of life” OR “terminally ill patient” OR “terminally ill”) 171,155

#3 TITLE-ABS-KEY  (aged OR elderly OR older OR “over 60” OR “over 60” OR “over 80” OR frail OR senior OR “very old” OR “old age” OR geriatric OR oldest OR elder OR “end of life” OR “late stage” OR “palliative care”) 6,608,502

#4 #1AND #2 AND #3 831

Filters: Publication years 2017 - 2019; English 139

Filter: article, article in press, review 129

**Cinahl Complete Update SWEDEN, 20170906 - 20190524**

S1 ( (MH "Palliative Care") OR (MH "Hospice and Palliative Nursing") OR (MH "Hospice Care") OR (MH "Hospices") OR (MH "Hospice Patients") OR (MH "Terminal Care+") OR (MH "Terminally Ill Patients+") ) OR ( “palliative nursing” OR “palliative therapy” OR “terminal care” OR hospice OR “hospice care” OR palliative OR “terminal illness” OR “palliative care” OR “palliative treatment” OR “palliative medicine” OR “end of life care” OR “end of life treatment” OR hospices OR “palliative intervention” OR “bereavement care” OR “end of life” OR “terminally ill patient” OR “terminally ill” ) 77,494

S2 (MH "Sweden") OR Sweden OR Swedish  32,802

S3 ( (MH "Aged+") OR (MH "Aged, 80 and Over") OR (MH "Frail Elderly") ) OR ( aged OR elderly OR older OR “over 60” OR “over 60” OR “over 80” OR frail OR senior OR “very old” OR “old age” OR geriatric OR oldest OR elder OR “end of life” OR “late stage” OR “palliative care” )  945,646

S4 S1 AND S2 AND S3 479

Filters: Published date: 20170701 - 20191231; English 67

**PsycInfo Update SWEDEN, 20170906 - 20190524**

S1 ( “palliative nursing” OR “palliative therapy” OR “terminal care” OR hospice OR “hospice care” OR palliative OR “terminal illness” OR “palliative care” OR “palliative treatment” OR “palliative medicine” OR “end of life care” OR “end of life treatment” OR hospices OR “palliative intervention” OR “bereavement care” OR “end of life” OR “terminally ill patient” OR “terminally ill” ) OR ( ((DE "Palliative Care") OR (DE "Hospice")) OR (DE "Terminally Ill Patients") )  38,967

S2 Sweden OR Swedish  62,189

S3 aged OR elderly OR older OR “over 60” OR “over 60” OR “over 80” OR frail OR senior OR “very old” OR “old age” OR geriatric OR oldest OR elder OR “end of life” OR “late stage” OR “palliative care”  721,882

S4 S1 AND S2 AND S3 718

Filters: 20170701 - 20191231; English 57

**Academic Search Complete Update SWEDEN, 20170906 - 20190524**

S1 ( ((DE "HOSPICE care" OR DE "HOSPICE nurses" OR DE "HOSPICE patients" OR DE "HOSPICES (Terminal care facilities)") OR (DE "PALLIATIVE treatment")) OR (DE "TERMINAL care") ) OR ( “palliative nursing” OR “palliative therapy” OR “terminal care” OR hospice OR “hospice care” OR palliative OR “terminal illness” OR “palliative care” OR “palliative treatment” OR “palliative medicine” OR “end of life care” OR “end of life treatment” OR hospices OR “palliative intervention” OR “bereavement care” OR “end of life” OR “terminally ill patient” OR “terminally ill” ) 138,483

S2 Sweden OR Swedish  300,744

S3 ( (DE "OLDER people") OR (DE "FRAIL elderly") ) OR ( aged OR elderly OR older OR “over 60” OR “over 60” OR “over 80” OR frail OR senior OR “very old” OR “old age” OR geriatric OR oldest OR elder OR “end of life” OR “late stage” OR “palliative care” )  1,071,525

S4 S1 AND S2 AND S3 1,163

Filters: 20170701 - 20191231; English 197

**Cochrane Library Update SWEDEN, 20170907 - 20190524**

## 'palliative OR hospice* OR "terminally ill" OR "end of life" (Title, Abstract, Keywords) AND Sweden OR Swedish (Title, Abstract, Keywords) 60

Filter: 2017 – 2019 13

**Updated Literature Searches – CHINA Palliative Care: Search strategies Sept 2017-2019**

**Embase Update CHINA, 20170906 - 20190531**

#1 'palliative nursing'/exp OR 'palliative therapy'/exp OR 'terminal care'/exp OR 'hospice'/exp OR 'hospice care'/exp OR palliative OR 'terminal illness' OR 'palliative care' OR 'palliative treatment' OR 'palliative medicine' OR 'terminal care' OR 'end of life care' OR 'end of life treatment' OR hospice OR hospices OR 'palliative intervention' OR 'end of life care'/exp OR 'bereavement care' OR 'end of life' 230,953

#2 #1 OR 'terminally ill patient'/exp OR 'terminally ill' 233,464

#3 aged OR elderly OR older OR 'over 60' OR 'over 60' OR 'over 80' OR frail OR senior OR 'very old' OR 'old age' OR geriatric OR oldest OR elder OR 'end of life' OR 'late stage' OR 'palliative care' OR 'aged'/exp 4,769,836

#4 'china'/exp OR 'chinese'/exp OR china OR Chinese 2,097,381
#5 #2 AND #3 AND #4 3,367

#6 #5 AND AND [english]/lim AND [1-9-2017]/sd NOT [3-6-2019]/sd 870

#7 #6 AND ('article'/it OR 'article in press'/it OR 'review'/it) 725

**PubMed Update CHINA, 20170906 - 20190531**

#1 "Palliative Care"[Mesh] OR "Palliative Medicine"[Mesh] OR palliative OR "terminal illness" OR "Terminal Care"[Mesh] OR "Hospice Care"[Mesh] OR "Hospices"[Mesh] OR "Hospice and Palliative Care Nursing"[Mesh] OR "palliative care" OR "palliative treatment" OR "palliative medicine" OR "terminal care" OR "end of life care" OR “end of life treatment” OR hospice OR hospices OR "palliative intervention" OR “terminally ill”[Mesh] OR “terminally ill” OR “end of life” OR “bereavement care” 149,294

#2 "China"[Mesh] OR China OR Chinese 1,699,533

#3 aged OR elderly OR older OR “over 60” OR “over 60” OR “over 80” OR frail OR senior OR “very old” OR “old age” OR geriatric OR oldest OR elder OR “end of life” OR “late stage” OR “palliative care” OR “aged”[Mesh] 5,414,611

#4 #1AND #2 AND #3 2,192

#5 Filters: Publication date from 2017/09/01 to present; English 371

Filter: journal article, review 362

## **Scopus Update CHINA, 20170906-20190531** #1 TITLE-ABS-KEY ( China OR Chinese )  1,329,281

#2 TITLE-ABS-KEY  (“palliative nursing” OR “palliative therapy” OR “terminal care” OR hospice OR “hospice care” OR palliative OR “terminal illness” OR “palliative care” OR “palliative treatment” OR “palliative medicine” OR “end of life care” OR “end of life treatment” OR hospices OR “palliative intervention” OR “bereavement care” OR “end of life” OR “terminally ill patient” OR “terminally ill”) 171,358

#3 TITLE-ABS-KEY  (aged OR elderly OR older OR “over 60” OR “over 60” OR “over 80” OR frail OR senior OR “very old” OR “old age” OR geriatric OR oldest OR elder OR “end of life” OR “late stage” OR “palliative care”) 6,666,203

#4 #1AND #2 AND #3 1,290

Filters: Publication years 2017 - 2019; English 287

Filter: article, article in press, review 260

**Cinahl Complete Update CHINA, 20170906-20190531**

S1 ( (MH "Palliative Care") OR (MH "Hospice and Palliative Nursing") OR (MH "Hospice Care") OR (MH "Hospices") OR (MH "Hospice Patients") OR (MH "Terminal Care+") OR (MH "Terminally Ill Patients+") ) OR ( “palliative nursing” OR “palliative therapy” OR “terminal care” OR hospice OR “hospice care” OR palliative OR “terminal illness” OR “palliative care” OR “palliative treatment” OR “palliative medicine” OR “end of life care” OR “end of life treatment” OR hospices OR “palliative intervention” OR “bereavement care” OR “end of life” OR “terminally ill patient” OR “terminally ill” ) 77,739

S2 (MH "China”) OR China OR Chinese 76,842

S3 ( (MH "Aged+") OR (MH "Aged, 80 and Over") OR (MH "Frail Elderly") ) OR ( aged OR elderly OR older OR “over 60” OR “over 60” OR “over 80” OR frail OR senior OR “very old” OR “old age” OR geriatric OR oldest OR elder OR “end of life” OR “late stage” OR “palliative care” )  950,836

S4 S1 AND S2 AND S3 398

Filters: Published date: 20170701 - 20191231; English 60

**PsycInfo Update CHINA, 20170906-20190531**

S1 ( “palliative nursing” OR “palliative therapy” OR “terminal care” OR hospice OR “hospice care” OR palliative OR “terminal illness” OR “palliative care” OR “palliative treatment” OR “palliative medicine” OR “end of life care” OR “end of life treatment” OR hospices OR “palliative intervention” OR “bereavement care” OR “end of life” OR “terminally ill patient” OR “terminally ill” ) OR ( ((DE "Palliative Care") OR (DE "Hospice")) OR (DE "Terminally Ill Patients") )  38,997

S2 China OR Chinese 114,832

S3 aged OR elderly OR older OR “over 60” OR “over 60” OR “over 80” OR frail OR senior OR “very old” OR “old age” OR geriatric OR oldest OR elder OR “end of life” OR “late stage” OR “palliative care”  722,239

S4 S1 AND S2 AND S3 616

Filters: 20170701 - 20191231; English 62

Filters: Academic Journals 57

**Academic Search Complete Update CHINA, 20170907-20190531**

S1 ( ((DE "HOSPICE care" OR DE "HOSPICE nurses" OR DE "HOSPICE patients" OR DE "HOSPICES (Terminal care facilities)") OR (DE "PALLIATIVE treatment")) OR (DE "TERMINAL care") ) OR ( “palliative nursing” OR “palliative therapy” OR “terminal care” OR hospice OR “hospice care” OR palliative OR “terminal illness” OR “palliative care” OR “palliative treatment” OR “palliative medicine” OR “end of life care” OR “end of life treatment” OR hospices OR “palliative intervention” OR “bereavement care” OR “end of life” OR “terminally ill patient” OR “terminally ill” ) 138,600

S2 China OR Chinese 2,259,775

S3 ( (DE "OLDER people") OR (DE "FRAIL elderly") ) OR ( aged OR elderly OR older OR “over 60” OR “over 60” OR “over 80” OR frail OR senior OR “very old” OR “old age” OR geriatric OR oldest OR elder OR “end of life” OR “late stage” OR “palliative care” )  1,072,605

S4 S1 AND S2 AND S3 1,873

Filters: 20170701 – 20191231; English 280

Filters: Academic Journals 272

**Cochrane Library Update China, 20170907-20190531**

## palliative OR hospice* OR "terminally ill" OR "end of life" (Title, Abstract, Keywords) AND China OR Chinese (Title, Abstract, Keywords) 100

## Filter: 2017-2019 24

**Original Literature Searches – SWEDEN Palliative Care: Search strategies Sept 2007-2017**

**Embase SWEDEN, 20170905**

((('sweden'/exp OR sweden OR swedish) AND (('palliative nursing'/exp OR 'palliative therapy'/exp OR 'terminal care'/exp OR 'hospice'/exp OR 'hospice care'/exp OR palliative OR 'terminal illness' OR 'palliative care' OR 'palliative treatment' OR 'palliative medicine' OR 'terminal care' OR 'end of life care' OR 'end of life treatment' OR hospice OR hospices OR 'palliative intervention' OR 'end of life care'/exp OR 'bereavement care' OR 'end of life') OR 'terminally ill patient'/exp OR 'terminally ill')
AND [english]/lim AND [2007-2017]/py) AND ('article'/it OR 'article in press'/it OR 'review'/it)) AND (aged OR elderly OR older OR 'over 60' OR 'over 60' OR 'over 80' OR frail OR senior OR 'very old' OR 'old age' OR geriatric OR oldest OR elder OR 'end of life' OR 'late stage' OR 'palliative care' OR 'aged'/exp) 1,040

#1 'sweden'/exp OR sweden OR Swedish 624,633

#2 'palliative nursing'/exp OR 'palliative therapy'/exp OR 'terminal care'/exp OR 'hospice'/exp OR 'hospice care'/exp OR palliative OR 'terminal illness' OR 'palliative care' OR 'palliative treatment' OR 'palliative medicine' OR 'terminal care' OR 'end of life care' OR 'end of life treatment' OR hospice OR hospices OR 'palliative intervention' OR 'end of life care'/exp OR 'bereavement care' OR 'end of life' 198,533

#3 #2 OR 'terminally ill patient'/exp OR 'terminally ill' 200,877

#4 #1 AND #3 AND [english]/lim AND [2007-2017]/py 2,475

#5 #4 AND ('article'/it OR 'article in press'/it OR 'review'/it) 1,495

#6 aged OR elderly OR older OR 'over 60' OR 'over 60' OR 'over 80' OR frail OR senior OR 'very old' OR 'old age' OR geriatric OR oldest OR elder OR 'end of life' OR 'late stage' OR 'palliative care' OR 'aged'/exp 4,168,399

#7 #5 AND #6 1,040

**PubMed SWEDEN, 20170905**

#1 "Palliative Care"[Mesh] OR "Palliative Medicine"[Mesh] OR palliative OR "terminal illness" OR "Terminal Care"[Mesh] OR "Hospice Care"[Mesh] OR "Hospices"[Mesh] OR "Hospice and Palliative Care Nursing"[Mesh] OR "palliative care" OR "palliative treatment" OR "palliative medicine" OR "terminal care" OR "end of life care" OR “end of life treatment” OR hospice OR hospices OR "palliative intervention" OR “terminally ill”[Mesh] OR “terminally ill” OR “end of life” OR “bereavement care” 132,904

#2 ("Sweden"[Mesh] OR Sweden OR Swedish)) 345,236

#3 aged OR elderly OR older OR “over 60” OR “over 60” OR “over 80” OR frail OR senior OR “very old” OR “old age” OR geriatric OR oldest OR elder OR “end of life” OR “late stage” OR “palliative care” OR “aged”[Mesh]

4,959,415

#4 #1AND #2 AND #3 1,475
Filters: Publication date from 2007/01/01 to 2017/12/31; English 827
Filter: journal article, review 813

## **Scopus SWEDEN, 20170906** #1 TITLE-ABS-KEY ( sweden  OR  swedish )  188,315

#2 TITLE-ABS-KEY  (“palliative nursing” OR “palliative therapy” OR “terminal care” OR hospice OR “hospice care” OR palliative OR “terminal illness” OR “palliative care” OR “palliative treatment” OR “palliative medicine” OR “end of life care” OR “end of life treatment” OR hospices OR “palliative intervention” OR “bereavement care” OR “end of life” OR “terminally ill patient” OR “terminally ill”) 154,692

#3 TITLE-ABS-KEY  (aged OR elderly OR older OR “over 60” OR “over 60” OR “over 80” OR frail OR senior OR “very old” OR “old age” OR geriatric OR oldest OR elder OR “end of life” OR “late stage” OR “palliative care”) 6,122,590

#4 #1AND #2 AND #3 745

Filters: Publication date from 2007/01/01 to 2017/12/31; English 416

Filter: article, article in press, review 388

**Cinahl Complete SWEDEN, 20170906**

S1 ( (MH "Palliative Care") OR (MH "Hospice and Palliative Nursing") OR (MH "Hospice Care") OR (MH "Hospices") OR (MH "Hospice Patients") OR (MH "Terminal Care+") OR (MH "Terminally Ill Patients+") ) OR ( “palliative nursing” OR “palliative therapy” OR “terminal care” OR hospice OR “hospice care” OR palliative OR “terminal illness” OR “palliative care” OR “palliative treatment” OR “palliative medicine” OR “end of life care” OR “end of life treatment” OR hospices OR “palliative intervention” OR “bereavement care” OR “end of life” OR “terminally ill patient” OR “terminally ill” ) 68,893

S2 (MH "Sweden") OR Sweden OR Swedish  28,861

S3 ( (MH "Aged+") OR (MH "Aged, 80 and Over") OR (MH "Frail Elderly") ) OR ( aged OR elderly OR older OR “over 60” OR “over 60” OR “over 80” OR frail OR senior OR “very old” OR “old age” OR geriatric OR oldest OR elder OR “end of life” OR “late stage” OR “palliative care” )  812,687

S4 S1 AND S2 AND S3 409

Filters: Publication date from 2007/01/01 to 2017/12/31; English 292

**PsycInfo SWEDEN, 20170906**

S1 ( “palliative nursing” OR “palliative therapy” OR “terminal care” OR hospice OR “hospice care” OR palliative OR “terminal illness” OR “palliative care” OR “palliative treatment” OR “palliative medicine” OR “end of life care” OR “end of life treatment” OR hospices OR “palliative intervention” OR “bereavement care” OR “end of life” OR “terminally ill patient” OR “terminally ill” ) OR ( ((DE "Palliative Care") OR (DE "Hospice")) OR (DE "Terminally Ill Patients") )  35,752

S2 Sweden OR Swedish  57,157

S3 aged OR elderly OR older OR “over 60” OR “over 60” OR “over 80” OR frail OR senior OR “very old” OR “old age” OR geriatric OR oldest OR elder OR “end of life” OR “late stage” OR “palliative care”  679,397

S4 S1 AND S2 AND S3 649

Filters: Publication date from 2007/01/01 to 2017/12/31; English 467

Filters: Journal Article, Review-Any 436

**Academic Search Complete SWEDEN, 20170906**

S1 ( ((DE "HOSPICE care" OR DE "HOSPICE nurses" OR DE "HOSPICE patients" OR DE "HOSPICES (Terminal care facilities)") OR (DE "PALLIATIVE treatment")) OR (DE "TERMINAL care") ) OR ( “palliative nursing” OR “palliative therapy” OR “terminal care” OR hospice OR “hospice care” OR palliative OR “terminal illness” OR “palliative care” OR “palliative treatment” OR “palliative medicine” OR “end of life care” OR “end of life treatment” OR hospices OR “palliative intervention” OR “bereavement care” OR “end of life” OR “terminally ill patient” OR “terminally ill” )

125,263

S2 Sweden OR Swedish  255,683

S3 ( (DE "OLDER people") OR (DE "FRAIL elderly") ) OR ( aged OR elderly OR older OR “over 60” OR “over 60” OR “over 80” OR frail OR senior OR “very old” OR “old age” OR geriatric OR oldest OR elder OR “end of life” OR “late stage” OR “palliative care” )  952,400

S4 S1 AND S2 AND S3 946

Filters: Publication date from 2007/01/01 to 2017/12/31; English 677

Filters: Journal Article, Peer-reviewed journals 614

**Cochrane Library SWEDEN, 20170907**

## 'palliative OR hospice* OR "terminally ill" OR "end of life" (Title, Abstract, Keywords) AND Sweden OR Swedish (Title, Abstract, Keywords) 41

## Filter: 2007-2017 24

**Original Literature Searches – CHINA Palliative Care: Search strategies Sept 2007-2017**

**Embase CHINA, 20170906**

#1 'palliative nursing'/exp OR 'palliative therapy'/exp OR 'terminal care'/exp OR 'hospice'/exp OR 'hospice care'/exp OR palliative OR 'terminal illness' OR 'palliative care' OR 'palliative treatment' OR 'palliative medicine' OR 'terminal care' OR 'end of life care' OR 'end of life treatment' OR hospice OR hospices OR 'palliative intervention' OR 'end of life care'/exp OR 'bereavement care' OR 'end of life' 198,592

#2 #1 OR 'terminally ill patient'/exp OR 'terminally ill' 200,937

#3 aged OR elderly OR older OR 'over 60' OR 'over 60' OR 'over 80' OR frail OR senior OR 'very old' OR 'old age' OR geriatric OR oldest OR elder OR 'end of life' OR 'late stage' OR 'palliative care' OR 'aged'/exp 4,169,957

#4 'china'/exp OR 'chinese'/exp OR china OR Chinese 1,691,957

#5 #2 AND #4 AND [english]/lim AND [2007-2017]/py 3,385

#6 #5 AND ('article'/it OR 'article in press'/it OR 'review'/it) 2,408

#7 #3 AND #6 1,440

**PubMed CHINA, 20170906**

#1 "Palliative Care"[Mesh] OR "Palliative Medicine"[Mesh] OR palliative OR "terminal illness" OR "Terminal Care"[Mesh] OR "Hospice Care"[Mesh] OR "Hospices"[Mesh] OR "Hospice and Palliative Care Nursing"[Mesh] OR "palliative care" OR "palliative treatment" OR "palliative medicine" OR "terminal care" OR "end of life care" OR “end of life treatment” OR hospice OR hospices OR "palliative intervention" OR “terminally ill”[Mesh] OR “terminally ill” OR “end of life” OR “bereavement care” 132,936

#2 "China"[Mesh] OR China OR Chinese 1,348,890

#3 aged OR elderly OR older OR “over 60” OR “over 60” OR “over 80” OR frail OR senior OR “very old” OR “old age” OR geriatric OR oldest OR elder OR “end of life” OR “late stage” OR “palliative care” OR “aged”[Mesh] 4,959,628

#4 #1AND #2 AND #3 1,746

Filters: Publication date from 2007/01/01 to 2017/12/31; English 1,259

Filter: journal article, review 1,233

## **Scopus CHINA, 20170906** #1 TITLE-ABS-KEY ( China OR Chinese )  1,093,076

#2 TITLE-ABS-KEY  (“palliative nursing” OR “palliative therapy” OR “terminal care” OR hospice OR “hospice care” OR palliative OR “terminal illness” OR “palliative care” OR “palliative treatment” OR “palliative medicine” OR “end of life care” OR “end of life treatment” OR hospices OR “palliative intervention” OR “bereavement care” OR “end of life” OR “terminally ill patient” OR “terminally ill”) 154,692

#3 TITLE-ABS-KEY  (aged OR elderly OR older OR “over 60” OR “over 60” OR “over 80” OR frail OR senior OR “very old” OR “old age” OR geriatric OR oldest OR elder OR “end of life” OR “late stage” OR “palliative care”) 6,122,590

#4 #1AND #2 AND #3 1,016

Filters: Publication date from 2007/01/01 to 2017/12/31; English 756

Filter: article, article in press, review 662

**Cinahl Complete CHINA, 20170906**

S1 ( (MH "Palliative Care") OR (MH "Hospice and Palliative Nursing") OR (MH "Hospice Care") OR (MH "Hospices") OR (MH "Hospice Patients") OR (MH "Terminal Care+") OR (MH "Terminally Ill Patients+") ) OR ( “palliative nursing” OR “palliative therapy” OR “terminal care” OR hospice OR “hospice care” OR palliative OR “terminal illness” OR “palliative care” OR “palliative treatment” OR “palliative medicine” OR “end of life care” OR “end of life treatment” OR hospices OR “palliative intervention” OR “bereavement care” OR “end of life” OR “terminally ill patient” OR “terminally ill” ) 68,893

S2 (MH "China”) OR China OR Chinese 59,816

S3 ( (MH "Aged+") OR (MH "Aged, 80 and Over") OR (MH "Frail Elderly") ) OR ( aged OR elderly OR older OR “over 60” OR “over 60” OR “over 80” OR frail OR senior OR “very old” OR “old age” OR geriatric OR oldest OR elder OR “end of life” OR “late stage” OR “palliative care” )  812,687

S4 S1 AND S2 AND S3 317

Filters: Publication date from 2007/01/01 to 2017/12/31; English 245

**PsycInfo CHINA, 20170906**

S1 ( “palliative nursing” OR “palliative therapy” OR “terminal care” OR hospice OR “hospice care” OR palliative OR “terminal illness” OR “palliative care” OR “palliative treatment” OR “palliative medicine” OR “end of life care” OR “end of life treatment” OR hospices OR “palliative intervention” OR “bereavement care” OR “end of life” OR “terminally ill patient” OR “terminally ill” ) OR ( ((DE "Palliative Care") OR (DE "Hospice")) OR (DE "Terminally Ill Patients") )  35,752

S2 China OR Chinese 99,132

S3 aged OR elderly OR older OR “over 60” OR “over 60” OR “over 80” OR frail OR senior OR “very old” OR “old age” OR geriatric OR oldest OR elder OR “end of life” OR “late stage” OR “palliative care”  679,397

S4 S1 AND S2 AND S3 541

Filters: Publication date from 2007/01/01 to 2017/12/31; English 393

Filters: Journal Article, Review-Any 351

**Academic Search Complete CHINA, 20170907**

S1 ( ((DE "HOSPICE care" OR DE "HOSPICE nurses" OR DE "HOSPICE patients" OR DE "HOSPICES (Terminal care facilities)") OR (DE "PALLIATIVE treatment")) OR (DE "TERMINAL care") ) OR ( “palliative nursing” OR “palliative therapy” OR “terminal care” OR hospice OR “hospice care” OR palliative OR “terminal illness” OR “palliative care” OR “palliative treatment” OR “palliative medicine” OR “end of life care” OR “end of life treatment” OR hospices OR “palliative intervention” OR “bereavement care” OR “end of life” OR “terminally ill patient” OR “terminally ill” )

125,280

S2 China OR Chinese 1,771,159

S3 ( (DE "OLDER people") OR (DE "FRAIL elderly") ) OR ( aged OR elderly OR older OR “over 60” OR “over 60” OR “over 80” OR frail OR senior OR “very old” OR “old age” OR geriatric OR oldest OR elder OR “end of life” OR “late stage” OR “palliative care” )  952,531

S4 S1 AND S2 AND S3 1,571

Filters: Publication date from 2007/01/01 to 2017/12/31; English 944

Filters: Journal Article, Peer-reviewed journals 715

**Cochrane Library CHINA, 20170907**

## palliative OR hospice* OR "terminally ill" OR "end of life" (Title, Abstract, Keywords) AND China OR Chinese (Title, Abstract, Keywords) 78

## Filter: 2007-2017 69
